# The Key Clinical Questions of Neoadjuvant Chemoradiotherapy for Resectable Esophageal Cancer—A Review

**DOI:** 10.3389/fonc.2022.890688

**Published:** 2022-07-14

**Authors:** Dan Han, Baosheng Li, Qian Zhao, Hongfu Sun, Jinling Dong, Shaoyu Hao, Wei Huang

**Affiliations:** ^1^ Shandong University Cancer Center, Jinan, China; ^2^ Department of Radiation Oncology, Shandong Cancer Hospital and Institute, Shandong First Medical University and Shandong Academy of Medical Sciences, Jinan, China; ^3^ Department of Thoracic Surgery, Shandong Cancer Hospital and Institute, Shandong First Medical University and Shandong Academy of Medical Sciences, Jinan, China

**Keywords:** esophageal cancer, neoadjuvant chemoradiotherapy, neoadjuvant immunotherapy, pathological complete remission (pCR), target volume delineation

## Abstract

Over 50% of individuals with esophageal cancer (EC) present with advanced stages of the disease; therefore, their outcome following surgery alone is poor, with only 25%–36% being alive 5 years post-surgery. Based on the evidence that the CROSS and NEOCRTEC5010 trials provided, neoadjuvant chemoradiotherapy (nCRT) is now the standard therapy for patients with locally advanced EC. However, there are still many concerning clinical questions that remain controversial such as radiation dose, appropriate patient selection, the design of the radiation field, the time interval between chemoradiotherapy (CRT) and surgery, and esophageal retention. With immune checkpoint inhibitors (ICIs) rapidly becoming a mainstay of cancer therapy, along with radiation, chemotherapy, and surgery, the combination mode of immunotherapy is also becoming a hot topic of discussion. Here, we try to provide constructive suggestions to answer the perplexing problems and clinical concerns for the progress of nCRT for EC in the future.

## Introduction

Over 50% of individuals with esophageal cancer (EC) present with advanced stages; therefore, their outcome following surgery alone is poor, with only 25%–36% being alive 5 years post-surgery (1). Based on current key evidence (2), preoperative chemoradiotherapy (CRT) or perioperative chemotherapy should be offered to patients with locally advanced esophageal adenocarcinoma (EAC), and preoperative CRT is the preferred mode of neoadjuvant therapy for patients with locally advanced esophageal squamous cell carcinoma (ESCC). Thus, a multidisciplinary approach is crucial for successful EC management (3). Using neoadjuvant chemoradiotherapy (nCRT) for EC management compared to surgery alone has been shown to provide overall survival (OS) benefits. This has led to increasing interest in approaches to optimize this treatment mode and to select appropriate patients.

## The Dose of Radiation Therapy

Currently, the dosage of radiation in the nCRT differs according to clinical trials and clinical experience with reports indicating that it varies from 20 Gy/10F to 50.4 Gy/28F (4–6). The National Comprehensive Cancer Network (NCCN) recommends radiation doses of 41.4–50.4 Gy for nCRT among patients with EC (7). In practice, a nationally representative survey of members of the American Society For Radiation Oncology (ASTRO) indicated that 50.4 Gy was the most common radiation dose used during nCRT of patients with EC in North America (8). The guideline for neoadjuvant radiotherapy of EC in China recommends 40–50.4 Gy (9), and 40–41.4 Gy is mostly used in China. A study (10) involving a national database with quality radiation records including its doses found no statistically significant difference in OS according to the neoadjuvant dose levels (40–41.4, 45, 50.4, and 54 Gy) regardless of histology after controlling for available confounding variables. A multi-institutional analysis (11) with 1,048 patients and a retrospective analysis with 118 patients (12) also concluded that there was an absence of radiation dose–response effect when compared to the pathological complete remission (pCR) rate. In both CROSS (4) and NEOCRTEC5010 (5) studies, lower radiation doses were used (41.4 Gy/23F or 40 Gy/20F), and this was associated with high efficacy and positive results, with pCR rate >40% and R0 rate >90%, suggesting that lower doses of radiotherapy of 40 Gy can also effectively kill tumor cells.

On the other hand, a retrospective single institution reported high radiation doses to be related to serious acute adverse effects (AEs) and poor conditions for surgical considerations (13). Defining the optimal radiation dose for nCRT in patients with EC requires the considerations of both benefits and potential AEs. This has not been evaluated in patients with EC who had nCRT followed by surgery. Based on the RTOG 9405 randomized controlled trial (RCT) (14), 50.4 Gy is now considered the standard dose for individuals with EC requiring concurrent chemoradiotherapy in European and American guidelines. What then is the significance of 50.4 Gy in neoadjuvant therapy? A higher radiation therapy dose was not significantly related to a higher pCR rate and longer survival. A lower dose might be a more appropriate time–dose fraction scheme. A meta-analysis (15) showed 48.85 Gy to be a biologically effective dose (BED), or 41.4 Gy in 23 fractions may be an adequate dose for nCRT treatment of patients with resectable EC and suggests creating treatment plans to 50.4 Gy with an intent to deliver 41.4 Gy. If clinical or radiographic assessment suggests that the patient is unfit to undergo surgery, then chemoradiation may be sustained to 50.4 Gy to deliver a definitive dose. It is important to note that nCRT is a technology used on a planned basis, which is different from radical CRT for the purpose of cure and conversion therapy for some patients. A matter of concern is that a lower radiation dose would be an inadequate definitive dose if such patients are subsequently unable to withstand the stress of surgery following neoadjuvant nCRT and in the era of using a wait-and-see policy in clinical complete responders. Thus, the scheme of nCRT itself still needs to be optimized and the selection for appropriate patient is the key point.

## The Details of Target Volume Delineation

Accurate estimation of the gross tumor volume (GTV) is needed during preoperative assessment of EC with nCRT. However, there is no gold standard definition of the irradiation volume and variability for tumor delineation can be large for EC. One prospective study (16) that analyzed the accuracy of GTV delineation and clinical target volume (CTV) margins for nCRT in EC at pathologic examination showed that a macroscopic tumor was located outside the GTV in 35% and outside the CTV in 14% of the patients with macroscopic residual tumors, which is associated with markedly worse OS. The mismatch of the GTV and macroscopic tumor indicates possible errors in the delineation.

The first concern is the delineation of the gross tumor volume of the primary tumor (GTVp) and the clinical target volume of the primary tumor (CTVp). GTVp includes the primary tumor also based on multi-modal image fusion including endoscopic ultrasonography (EUS), computerized tomography (CT) scan, magnetic resonance imaging (MRI), and fluorodeoxyglucose positron-emission tomography (FDG-PET), and endoscopic fiducial markers should be included. In the European Society for Radiation Oncology (ESTRO) proposal (17), “CTVp includes the GTVp with an expansion of 1.0 cm radially and 3.0 cm cranio-caudally along the esophageal wall. For tumours in the lower esophagus and gastro-esophageal junction (GEJ), the CTVp is restricted to 2.0 cm distal to the tumour”. The NEOCRTEC5010 trial (5) and the CROSS study (4) supported the above views of CTVp. A pathological analysis (18) concluded that a 3.0-cm longitudinal margin from GTVp to CTVp may be adequate for the majority of cases of EC within the esophagus, but for the distal margin of GEJ adenocarcinoma, a 5.0-cm longitudinal margin from gross disease to CTV is needed to cover microscopic disease in 94% of cases. However, due to the existence of surgery, some experts suggest whether it is necessary to greatly enlarge the cranio-caudal margin and whether it can only be put out 2 cm, or even 1 cm.

Lymph node involvement in EC has a great impact on both target volume delineation and prognosis of the patients. The second concern is how to delineate such a pathological lymph node and the area of subclinical involvement of EC patients. The volume of the pathological lymph nodes (GTVn) includes the involved lymph nodes being considered pathological based on the multi-modal image fusion any time before the radiation therapy. The use of EUS-guided fine-needle aspiration cytology (FNAC), which will increase the accuracy of regional lymph node to approximately 85% (19), is recommended in case of doubt and when it is associated with the delineation of the target volume. In the ESTRO proposal (17), the clinical target volume of the nodes (CTVn) includes the GTVn with an increase in 1.0 cm in all directions, and the involved lymph node stations, including the vena azygos, the aortic-pulmonal fenestra, and the fatty tissue of the arteria gastrica sinistra and of the subcarinal, para/pretracheal, paracardial, and supraclavicular region as long as they are up to 3.0 cm cranio-caudally from GTVp, should be additionally irradiated. However, we believe that the target area involved above is too large, and they may adversely affect treatment complication risks. Radiotherapy is well known to have a lymphocyte killing effect and can destroy mature circulating lymphocytes. A study (20) observed that the tissue receiving radiotherapy would theoretically spare tumor-associated lymphocytes from the peri-tumoral tumor microenvironment, which can be rich in immune cells and can contain tertiary lymphoid structures. Radiation-induced lymphocyte killing has been reported to be adversely associated with poorer OS and progression-free survival (PFS) (21). A similar study (22) from the MD Anderson Cancer Center reported that grade 4 lymphopenia was significantly correlated with poorer OS and PFS in 272 EC patients who received nCRT. Especially in combination with immunotherapy, excessive irradiation of normal lymph nodes will affect the release of effector T cells, thus affecting the efficacy.

Wang et al. (23) assessed 217 individuals with ESCC, and proposed a margin of 3.0–5.0 mm from GTVn to CTVn in order to include 95% of the extracapsular extension of lymph node, which depends on the diameter of the lymph node. CTVn provided a 0.5- to 1.0-cm radial margin around the GTVn to include the area of subclinical involvement in the NEOCRTEC5010 trial (5). If there is no invasion, the CTVn is corrected for anatomy barrier such as muscles and bones.

The third concern is whether radiotherapy should adopt involved-field irradiation (IFI) or elective nodal irradiation (ENI). The CROSS trial (24) with IFI using three-dimensional conformal radiotherapy (3D-CRT) reported infield recurrences in 11 (5.2%) of 213 patients, with only two patients having an infield recurrence without synchronous distant failure and recurrences at the borders of the treatment volume occurred in five (2.3%) of 213 patients, and regional outfield recurrences occurred in thirteen (6.1%) of 213 patients. In the CROSS trial, histology was mostly adenocarcinoma (75%). In a review investigating the pattern of recurrences and involving 23 non-randomized trials of individuals who received preoperative nCRT, locoregional, distant, and total recurrence rates ranged between 0% and 39%, 19% and 70%, and 19% and 80%, respectively (25). Thoen et al. (26) also confirmed that distant failure is the most common mode of failure in individuals having EC with 39% locoregional recurrence rates and 59% distant recurrence rates. HSU et al. (27) retrospectively studied 118 patients, of whom 73 patients with ENI were given radiotherapy to either supraclavicular (*n* = 54) or celiac (*n* = 19) lymphatics and concluded that preoperative nCRT followed by surgery was not associated with survival benefits and did not improve disease control for ESCC. Omission of ENI was related to higher M1a failures, but did not increase the isolated distant nodal failure. A retrospective study (28) involving 222 patients (111 matched pairs treated with IFI versus ENI) with nonmetastatic GEJ carcinomas treated with concurrent chemoradiation ± surgery, in which the ENI additionally included the celiac and splenic (± porta) lymph nodes, concluded that no patients failed in the splenic or porta nodes. A meta-analysis (29) of twenty-nine RCTs with a total of 5,212 patients concluded that no significant differences in locoregional recurrence, OS, R0 resection distant metastases, and postoperative mortality were observed between IFI and ENI. In subgroup analyses, IFI had a statistically significant OS advantage over nCRT for ESCC, and ENI appears to be more effective for individuals with EAC. As a consequence, it is unlikely to increase survival with efforts to improve locoregional control such as extensive lymphadenectomy or extension of the radiation volume. Currently, most studies support the IFI in esophageal neoadjuvant therapy due to the low field recurrence rate. However, some experts hold the opposite view. Two expert panels in two articles (30, 31) proposed delineation of ENI stations according to the primary tumor location in the nCRT setting in EC. We believe that individualized delineation is needed according to tumor location and pathologic type.

The effects of neoadjuvant radiation on postoperative anastomotic leaks is noteworthy, and the last concern is how to design the radiation field. A retrospectively study (32) of 285 EC patients treated with nCRT of 50.4 Gy at 1.8 Gy per fraction IFI showed that the anastomotic location relative to the field of radiation is a crucial factor affecting the occurrence of postoperative leaks after esophagectomy with an anastomotic leak rate of 31.8% in patients whose anastomosis was done inside the radiation field compared with 7% in patients whose anastomoses were placed outside the radiation field (*p* < 0.0001). The upper boundary of the target area does not exceed the clavicular head level in the NEOCRTEC5010 trial (5). Surgeons should cautiously assess and exclude individuals with previously irradiated esophagus and stomach when creating the esophagogastric anastomosis. Other factors found to be associated with anastomotic leaks include the tumor location, the type of surgery, and diabetes.

More details regarding how the volume was estimated and radiation field coverage are not yet studied using RCTs. In general, IFI is widely used in clinical studies and practical work in China, and further confirmation is needed from data in phase III multicenter randomized controlled studies.

## The Choice/Optimizationof Concurrent Chemotherapy Regimen

Cisplatin and 5-fluorouracil (PF)-based CRT regimens have been commonly used as neoadjuvant therapy for EC in the past few years. The response rate for neoadjuvant PF in the JCOG9907 study (33) was restricted to 38%, and subgroup analysis indicated that neoadjuvant therapy using the PF scheme failed to benefit cohorts with clinical stage III ESCC, indicating that a more effective regimen is required for individuals with clinical stage III ESCC. Since 2012, when the CROSS (3) trial was published, many centers have changed their standard neoadjuvant chemotherapy scheme to carboplatin/paclitaxel. A meta-analysis of 31 clinical studies (34) concluded that taxane-based therapy and PF had equivalent efficacy and taxane-based therapy had better OS (nCRT: pooled HR = 0.51, *p* = 0.03) than PF chemotherapy in ESCC patients. However, a new propensity score-matched study (35) that is divided into a PFRT Group (neoadjuvant PF with 40 Gy radiation dose in 20 daily fractions) and a CROSS Group (carboplatin/paclitaxel with 41.4 Gy radiotherapy in 23 daily fractions) from 2002 to 2019 concluded that no statistically significant differences exist in both groups in terms of survival or their clinical pathological outcome in ESCC patients, but the trend favors the PF scheme.

The NEOCRTEC5010 study (5) used the vinorelbine/cisplatin (NP) scheme and also obtained a good tolerance and effectiveness in China ESCC patients. A retrospective analysis (36) further pointed out that compared with the VP2 scheme (cisplatin 25 mg/m^2^ on days 1 to 4, and vinorelbine 25 mg/m^2^ on days 1 and 8), VP1 (cisplatin 75 mg/m^2^ on day 1, and vinorelbine 25 mg/m^2^ on days 1 and 8) showed comparable effectiveness considering survival benefits, lower hematologic toxicity, and postoperative pulmonary infection.

Other triplet chemotherapy regimens are also under clinical study. A phase II trial (37) that enrolled 28 patients showed that combination chemotherapy with DNF (docetaxel, nedaplatin, and 5‐fluorouracil) is a promising scheme for resectable EC, having acceptable feasibility with a 89.3% completion rate for protocol treatment. Preoperative chemotherapy with the triplet scheme of albumin-bound paclitaxel, cisplatin, and capecitabine for those having locally advanced ESCC also showed substantial tumor reduction and an encouraging pCR rate, with less toxicities in a retrospective study (38).

In terms of AEs, the addition of two-drug chemotherapy was tolerable, and there was no increase in postoperative morbidity (39, 40). Three-drug therapy showed a higher rate of grade 3/4 toxicities than what has been previously observed with two-drug chemotherapy, especially in hematological components (41). The pattern of preoperative pembrolizumab combined with chemoradiotherapy (PPCT)-related (42) postoperative infectious complications was not low with pneumonia (4.22%) and atelectasis (4.22%). Postoperative morbidity is closely related to esophagectomy methods and less invasive operation may be beneficial to patient outcome. The preliminary results need further confirmation from larger samples. Postoperative adjuvant chemotherapy is recommended for EAC and GEJ adenocarcinoma (43), while adjuvant chemotherapy for ESCC improves disease-free survival (DFS) but does not improve OS (44, 45).

As a whole, the cisplatin-based treatment regimen is the most commonly used regimen in EC neoadjuvant therapy. The selection of chemotherapy regimens should be individualized, combined with effectiveness and toxicity.

## The Optimal Interval After Neoadjuvant Chemoradiotherapy

The optimal time between nCRT and surgery for individuals with EC is not clear. Patients were suggested in the nCRT plus surgery arm to have the surgical procedure after an interval of 6 weeks in the CROSS study and after an interval of 4 to 6 weeks in the NEOCRTEC5010 study after completion of nCRT. A study (46) included 2,444 patients’ data that were collected from the National Cancer Database (NCDB), and demonstrated that surgical resection for EC within 56 days of neoadjuvant therapy is related to improved OS. Also, in prospective randomized controlled clinical studies, it is widely agreed that esophagectomy should be performed after a period of 6–8 weeks. However, in a retrospective study (47) involving 266 individuals with EC, Kim et al. explored the effectiveness of the interval between nCRT and esophagectomy, and compared those with shorter than 8 weeks versus intervals of 8 weeks or longer and found no significant difference in surgical morbidity, pCR rate, or OS between the two groups. Another study (48) divided patients into short-interval (<39 days) and long-interval (≥39 days) groups based on the interval between nCRT and surgery for EC and also showed that prolonging the interval had no effect on pCR rates or survival, but substantially increased the risk of postoperative complications, like anastomotic leakage and recurrent laryngeal nerve palsy. Prolonging the interval may lead to the surgical procedure being more challenging due to the radiation-induced fibrosis, which may make some locoregional residual disease progress to an unresectable stage, potentially affecting OS.

Delaying the operation interval after completion of nCRT may also increase concerns about regrowth of primary or metastatic tumors in EC. Chien et al. (49) found that unfavorable pathological descriptors such as closer circumferential resection margin and non-R0 resection were more observed after an interval of more than 8 weeks between nCRT and surgery. They further reported that the 5-year OS in patients with cCR to nCRT declined to 35% as opposed to 50% in the patients who underwent surgery within 8 weeks (*p* = 0.038). Although there are concerns that tumor progression occurred after excessive operation delays, this is difficult to confirm because there are no standardized criteria for assessing tumor progression after nCRT. Causality could not be evaluated, and this issue will require prospective randomized trial evaluation.

However, some scholars hold a different view that CRT induces cell death, by stimulating an immune response specific to the malignancy; this improves immune surveillance and tumoricidal capacity for many months after nCRT completion, extending surgical timing beyond 6 to 8 weeks (50, 51). Haisley et al. (52) suggest that an nCRT completion interval of 85 to 98 days before the surgical resection is significantly related to a higher odds of a pCR in individuals with EC. The observation of higher pCR rates with longer time intervals between the nCRT completion and esophagectomy may be suggestive of the increased tumoricidal capacity in the post-neoadjuvant therapy setting. The study further indicated that conducting esophagectomy within the currently recommended time frame (6–8 weeks) may refocus the immune system’s capability toward recovering from a major surgical operation rather than continuing in its tumoricidal role. However, the time to immune recovery in the weeks following nCRT needs to be more formally assessed, and it is vital to screen patients with poor or no response to CRT based on intrinsic tumor resistance.

## The Age Limit of Neoadjuvant Chemoradiotherapy

The number of elderly patients with EC increases with the increasing aging world population and the increasing worldwide actuarial life expectancy. Miyata et al. (53) concluded that elderly EC patients aged 75 years and greater, especially octogenarians, have a poorer prognosis than younger patients partly because they less often received neoadjuvant therapy. The upper age limit of neoadjuvant therapy for EC has not been determined. In many clinical studies, individuals having poorer performance status and the elderly were not enrolled; the upper age limit was 70 in the NEOCRTEC5010 trial (5) and 73 in the CROSS study (4). The usefulness of nCRT combined with esophagectomy therapy to these elderly patients requires further investigation.

With the technology of radiotherapy, minimally invasive anastomosis, perioperative management, rapid rehabilitation, and so on becoming more mature, aggressive therapy should be selected in order to achieve favorable prognosis based on the physical condition of elderly patients. We also need adequate multidisciplinary assessment to understand the negative impact of neoadjuvant therapy on elderly patients.

## Whether to Take a “Wait-and-See” Policy After Neoadjuvant Chemoradiotherapy or Not

According to the NCCN guidelines, no consensus was reached on whether a trimodality approach was superior to CRT alone in individuals with resectable EC. In practical clinical practice, not all patients with EC who receive nCRT can finally complete surgical resection as planned for a variety of reasons such as poor health, unexpected distant metastases, and refusal of surgery after nCRT. So what is the next step? We tried to find some answers from the literature. Firstly, a study (54) included 431 participants in an RCT evaluating CRT plus esophagectomy versus CRT only for individuals with localized EC. Their finding suggested that moderate-quality evidence was found affirming that inclusion of esophagectomy to CRT most likely lowered the risk of locoregional relapse (HR 0.55; 95% CI: 0.39–0.76, *p* = 0.0004). However, low-quality evidence indicated a higher risk of treatment-related mortality (RR 5.11; 95% CI: 1.74–15.02, *p* = 0.003), and high-quality evidence showed no OS benefits (HR 0.99; 95% CI 0.79–1.24, *p* = 0.92). Secondly, Rawat et al. identified individuals who had concurrent CRT (50 Gy, 40 mg/m^2^ of cisplatin per week) and assessed the therapeutic effect after 6 weeks. Nineteen individuals with resectable tumors had surgery, while others were considered as the observation group (active surveillance). No significant statistical differences in OS or PFS were observed between the groups (55).

However, it is commonly understood that individuals who are responsive to induction therapy tend to have a better prognosis (54). For these patients, after the induction therapy regimen was used as active surveillance, surgical procedures or CRT is still riddled with controversies. The difficulty lies in how to identify these patients. Jeong et al. (56) classified ESCC patients with complete clinical response (cCR) that was evaluated by FDG-PET, which, after CRT and surgery versus definitive CRT groups, showed that the surgery group had an advantage over the definitive CRT group in 2-year OS, local recurrence-free survival (LRFS), and PFS. However, Castoro et al. (57) carried out a similar study and revealed no statistical differences in 5-year OS and PFS between surgery and active surveillance groups with cCR that were evaluated upon endoscopic observation of the entire esophagus after nCRT. A meta-analysis (58) from China concluded that the inclusion of esophagectomy in individuals with cCR after CRT for thoracic locally advanced EC had no advantage on OS, while 2-year PFS showed some improvements. It is noteworthy that the diagnosis of cCR does not follow any standardized approach globally, and cCR itself does not accurately demonstrate the occurrence of pCR. This may account for the inconsistent conclusions of the above studies.

If patients with cCR after nCRT give up radical surgery, or for whatever reason miss the optimal interval time, will they still have the opportunity to undergo salvage surgery in the future once they relapse? A large retrospective database study (59) enrolled 8,489 patients with EAC from 2004 to 2014 who had preoperative nCRT and esophagectomy. Subjects who had their surgeries less than 90 days after nCRT were classified as the timely esophagectomy group (*n* = 7,822), while those who had their surgery over 90 days after nCRT were named as the delayed esophagectomy group (*n* = 667). The conclusion was that there was no significant difference in long-term survival among the subjects who delayed esophagectomy for adenocarcinoma compared to the timely esophagectomy group. Thus, delayed and salvage esophagectomy can be given to individuals who did not get timely esophagectomy after nCRT.

Esophagectomy is associated with a long-standing effect on health-related life quality. Patients with EC who completed questionnaires 4–6 weeks after nCRT were willing to trade off 16% 5-year OS to lower the risk of esophagectomy that is necessary from 100% to 35% in a prospective discrete-choice experiment (60). Controversy remains over whether an active surveillance strategy should be applied to patients with a cCR after nCRT for EC. Theoretically, active surveillance may be a useful approach in subjects without locoregional or disseminated disease, given that esophagectomy probably does not affect outcomes in patients with no viable tumor cells. The Dutch Surgery As Needed for esophageal cancer (SANO) trial and the French ESOSTRATE trial are under way to investigate the necessity for surgery in patients who achieve cCR after neoadjuvant therapy (61, 62). In the future, some patients might avoid surgery and thus have a higher quality of life with entire functioning organs on the premise of locoregional control.

## The Strategies to Accurately Predict the Efficacy of Neoadjuvant Chemoradiotherapy

Accurate prediction of the pCR before surgery is crucial for the decision of whether to continue observation or radical surgery in the observation period after nCRT. A single-center retrospective study (63) with 146 ESCC patients treated with nCRT concluded that a ≤40% reduction in the maximal esophageal wall thickness following nCRT was strongly related to low pCR rate, short survival time, and high risk of recurrence. However, the prediction of the efficacy of nCRT-based CT alone is insufficient. A prospective cohort study (64) that enrolled 138 patients concluded that the prediction of pCR through endoscopy and PET-CT independently or combined is subjected to low sensitivity and poor positive predictive value. A meta-analysis (65) involved 44 studies assessing the accuracy of EUS, endoscopic biopsies, or PET-CT for diagnosing locoregional residual disease after nCRT for squamous cell carcinoma or adenocarcinoma, which also showed that the accuracy is insufficient. Therefore, protocols to minimize surgery in subjects with apparent cCR based on PET-CT and/or endoscopic biopsies should be adopted with considerable caution. A prospective, multicenter, and diagnostic cohort preSANO study (61) at six centers in the Netherlands aimed to establish the accuracy of residual disease detection after nCRT in individuals with EC or GEJ cancer, as reflected by the percentage of tumors classified as tumor regression grade (TRG) 3 or TRG 4 that was missed during clinical response evaluations, and recommended that clinical response evaluations should include fine-needle aspiration of suspicious lymph nodes and repeated endoscopy with bite-on-bite biopsies for the detection of locoregional residual disease and PET/CT for the discovery of interval metastases. However, for this optimal combination of modalities for detecting response, a tumor regression grade of 3 or 4 was missed in 10% of cases. Borggreve et al. (66) enrolled 24 patients during a period of 2.5 years and revealed that treatment-induced change in tumor apparent diffusion coefficient (ADC) as measured on diffusion-weighted magnetic resonance imaging (DW-MRI) during the second week is most predictive for pCR to nCRT in ESCC and EAC. However, the relatively small study sample might have led to false-negative results (type II error) and DW-MRI scanning is currently not regularly utilized in the staging of patients with EC, which challenges the direct implementation of the study findings in clinical practice. 18F-FDG PET/CT and DW-MRI might be of complementary value in the assessment of pCR. Borggreve et al. (67) also conducted a prospective multicenter study and further concluded that changes on 18F-FDG PET/CT after nCRT and early changes on DW-MRI during nCRT may be useful for detecting pCR to nCRT in EC. Dynamic contrast-enhanced MRI and DW-MRI are emerging techniques that hold promise and need to be evaluated in future bigger diagnostic trials.

In addition, the constructed models are a meaningful step in locally advanced EC for predicting response to nCRT, and some studies aim at developing a multimodal clinically applicable prediction model. Fu Jianhua et al. (68) developed and validated a model using ResNet50 that contained 14 features and reached the best classification performance when comparing the six models adopting different convolutional neural networks as a feature extractor based on the deep learning or the handcrafted radiomics methods respectively. Roelof et al. (69) added the 18F-FDG PET-derived PET textural feature long-run low gray-level emphasis (LRLGLe-PET) and CT textural feature run percentage (RP-CT) to construct a predictive model with the clinical parameter histologic type and clinical T-stage. The predictive values of the constructed models were more accurate than response prediction based on SUVmax. Further studies are needed to revalidate the predictive value of these models to avoid surgery in selected cases.

So far, no clinically available noninvasive biomarkers can predict pCR for EC with nCRT. Roelof (70) indicated that the combination of human epidermal growth factor receptor 2 (HER2) and cluster of differentiation 44 (CD44) into 18F-FDG PET-based clinico-radiomic feature (Geary’s C measure and long-run low gray-level emphasis) prediction models improved nCRT response prediction in EC through assessing the expression of HER2 and CD44 by immunohistochemistry in pre-treatment tumor biopsies of 96 subjects. Currently, with the developments in high-throughput sequencing technology, multiple messenger RNAs (mRNAs) or microRNAs (miRNAs) were especially validated as useful biomarkers, able to relate the PCR of ESCC to nCRT. Jie He et al. (71) have demonstrated a novel three-long noncoding RNA (lncRNA-based) corresponding statistical model using a large number of endoscopic cancer biopsies obtained from ESCC subjects before treatment to determine the pathological response and outcome with nCRT. After an examination of immune-specific signatures from pretreatment endoscopic samples taken from pCRs and less than pCRs, Jie He et al. (72) recruited four immune-related genes—Serpin Family E Member 1 (SERPINE1), matrix metalloproteinase-12 (MMP12), urokinase type plasminogen activator receptor (PLAUR), and epidermal growth factor receptor kinase substrate 8 (EPS8)—for pCR and outcome prediction of ESCC through a multicenter analysis. However, this research was a retrospective cohort study from different institutions based on formalin-fixed paraffin-embedded samples. Future prospective studies should examine fresh samples. Based on the microarray datasets of nCRT containing both the responder and non-responder samples (accession numbers GSE45670 and GSE59974) of individuals with ESCC that were obtained from the Gene Expression Omnibus (GEO) database, Wang et al. (73) also identified that abnormal expression of MMP12 was significantly related to pathological degree, TNM stage, lymph nodes metastasis, and OS of ESCC patients (*p* < 0.05).

Such a series of methods would allow clinicians to use early intervention and switch to another therapeutic schedule if patients could not pathologically completely respond to nCRT. It may be worthwhile to select the subjects having a favorable molecular signature for treatment response and/or resistance to CRT.

## Combination of Anti-PD-1/PD-L1 Therapy

It is well known that nCRT is closely related to the immunogenetic changes of tumor and the tumor microenvironment in EC. Programmed cell death-ligand 1 (PD-L1) was overexpressed in 43.7% of ESCC patients (74). A study (75) revealed that a positive PD-L1 expression is related to poor response to CRT and poor survival of patients with ESCC receiving esophagectomy after nCRT. Recently, another study (76) has also revealed that immune checkpoints such as indoleamine 2,3-dioxygenase 1 and PD-L1 co-expression could identify subjects with poor pathologic response and those having high risk of recurrence in ESCC after nCRT, suggesting that some patients may benefit from CRT combined with anti-PD-1/PD-L1 therapy.

Immune checkpoint inhibitors (ICIs) are increasingly becoming a mainstay of cancer therapy, along with radiation, chemotherapy, and surgery. Smita et al. (77) retrospectively identified patients with locally advanced EC who received nCRT and immunotherapy (*n* = 25) versus those who received CRT alone (*n* =143) and concluded that overall rates of 30-day mortality and readmission did not significantly differ in patients treated with neoadjuvant immunotherapy (0% vs. 1.4%, 17% vs. 13%). The combination of nCRT and immunotherapy was safe. To further investigate the tolerability and efficacy of PPCT for resectable ESCC, Li et al. firstly conducted a prospective PALACE-1 trial (42) that included 20 resectable ESCC patients, regardless of PDL-1 status, who received a preoperative PPCT pattern, and concluded that PPCT-related AEs (any grade) were similar to nCRT. The most common grade III and higher AE was lymphopenia, and one patient developed a grade V AE due to esophageal hemorrhage. Indeed, PPCT was safe, did not delay surgery, and induced a pCR in 55.6% of resected tumors. The combination of neoadjuvant immunotherapy and CRT model has shown benefits in ESCC, but what about the combined efficacy in EAC? The phase II PERFECT trial (78) enrolled 40 resectable EAC patients who received nCRT based on the CROSS regimen combined with five cycles of atezolizumab (1,200 mg) and concluded that the pCR rate was higher than the CROSS trial (pCR 30% vs. 23%) and immune-elated AEs of any grade were observed in six patients. Compared to a propensity-matched cohort treated with nCRT pathological response, no statistically significant difference in response or survival was found between the PERFECT and the nCRT cohort. Furthermore, they further indicated that the expression of an IFNγ signature was related to response.

Preliminary findings of phase II clinical trials exploring the combination of ICIs with nCRT in EC showed encouraging efficacy with manageable toxicity. In order to reduce the side effects and improve the quality of neoadjuvant therapy in patients with EC, some scholars put forward the idea of replacing chemotherapy with immunotherapy. Our cancer center is conducting an open, single-center, phase Ib clinical trial (79) for assessing radiotherapy and toripalimab for neoadjuvant treatment of resectable ESCC. The trial revealed that neoadjuvant radiotherapy plus toripalimab showed an acceptable safety profile and a promising therapeutic effect with (47.4%, 9/19) experienced pCR (ypT0N0) of primary tumor and lymph nodes. We look forward to more prospective clinical trials to find out whether this combination of radiotherapy and immunotherapy for ESCC is effective and feasible.

In addition, many studies (80–83) were interested to see how EC would respond to the combination of neoadjuvant immunotherapy plus chemotherapy. A phase II study (82) enrolled 56 resectable locally advanced ESCC patients in whom preoperative camrelizumab plus nab-paclitaxel-cisplatin has an encouraging pCR of 35.3% and a manageable safety profile. A similar study (83) that involved 23 resectable ESCC patients concluded that neoadjuvant camrelizumab with nab-paclitaxel and carboplatin had tolerable treatment-related AEs and received an objective response of 90.5%, providing a feasible neoadjuvant option for these patients. Prospective clinical trials are needed to confirm the feasibility of this combination model in terms of getting rid of radiotherapy and whether there is a difference between squamous and adenocarcinoma.

Currently, undergoing surgery after nCRT appears to be the gold standard approach to managing patients with resectable EC. However, recurrence risk after nCRT and surgery remains high, especially among subjects without a pCR. The global phase III CheckMate 577 trial (84) enrolled 794 patients with resected (R0) stage II or III esophageal or gastroesophageal junction cancer who had received nCRT and not achieved pCR and who were randomly assigned in a 2:1 ratio to receive nivolumab or matching placebo, and concluded that the median DFS was 22.4 months among the patients who received nivolumab as compared with 11.0 months among the patients who received placebo. In patients who received nivolumab, the similar hazard ratios for disease recurrence or death with tumor cell PD-L1 expression either below 1% or 1% or higher and the magnitude of benefit with respect to DFS were higher in those in whom nivolumab was given at 10 weeks or more following surgery compared to those in whom nivolumab was initiated less than 10 weeks after surgery.

These previous findings and ongoing studies (**Table 1**) showed the potential for a combination of immunological therapy in locally advanced EC patients. We look forward to more phase III clinical trials to answer the question of which combination is optimal.

**Table 1:** Summary of ongoing neoadjuvant immunotherapy studies for locally advanced esophageal cancer.

Estimated Start–End Date	Identifier	Phase	n	Histology	Treatment Strategy	Primary Endpoint
March 2017–April 2025	NCT02998268	II	46	EAC	Arm1 : Pembro+CRT→Surgery→Pembro Arm2 : Pembro+CT→Surgery→Pembro	DFS rate (1 year)
May 2021–May 2028	Keystone - 002 NCT04807673	III	342	ESCC	Arm1 : Pembro+CRT→Surgery→Pembro Arm2 : Pembro+CT→Surgery→Pembro	DFS rate (2.5 years)
July 2020–December 2024	Keystone - 001NCT04389177	II	50	ESCC	Pembro+CT→Surgery→Pembro	MPR
August 2020–June 2025	PALACE-2 NCT04435197	II	143	ESCC	Pembro+CRT→Surgery→Pembro	pCR
October 2017–May 2023	PROCEED NCT03064490	II	38	EGC	Pembro+CRT→Surgery→Pembro	pCR
October 2019–February 2025	NCT04089904	II	33	EAC, EGC, GC	Pembro→Surgery	pCR
April 2018–December 2040	INEC NCT03544736	II	30	EC	Arm1 : Nivo+RT→Nivo Arm2 : Nivo+CRT→Nivo Arm3 : Nivo+CRT→Surgery→Nivo	Safety and Tolerability
August 2019–July 2022	NCT03987815	II	20	ESCC	Nivo→Surgery	MPR
June 2017–June 2021	NCT03278626	I	10	ESCC	Nivo+CRT→Surgery	Safety and Tolerability
February 2018–December 2023	NCT03288350	II	55	EGA	Avelumab+CT→Surgery	pCR
May 2018–February 2024	NCT03490292	II	24	EGA	Avelumab+CRT→Surgery	Dose-Limiting Toxicity; pCR
November 2016–November 2023	NCT02962063	II	78	EGA, EAC	Durvalumab +Tremelimumab+CRT→Surgery	Tolerability;pCR
May 2020–June 2022	NCT04221555	II	68	EGC, GC	Durvalumab +CT→Surgery→Durvalumab	pCR
June 2020–December 2023	NCT04568200	II	60	ESCC	Arm1 : Durvaluma+CRT→Surgery Arm2 : Placebo+CRT→Surgery	ORR
March 2021–March 2023	NCT04767295	II	28	ESCC	Camrelizumab+CT→Surgery	pCR
August 2020–December 2025	NCT04506138	II	46	ESCC	Camrelizumab+CT→Surgery	pCR
May 2019–October 2022	KEEP-G 03 NCT03946969	II	30	ESCC	Sintilimab+CT→Surgery	Safety and Tolerability
April 2021–March 2024	NCT04804696	II	53	ESCC	Toripalimab+CT→Surgery	pCR
September 2020–March 2024	NCT04644250	II	44	ESCC	Toripalimab+CT→Surgery	pCR
July 2020–December 2023	NCT04437212	II	20	ESCC	Toripalimab+CRT→Surgery	MPR
March 2020–December 2023	NCT04177797	II	20	ESCC	Toripalimab→Surgery	PCR
June 2019–December 31, 2020	NCT04006041	II	44	ESCC	Toripalimab+CRT→Surgery	PCR
June 2021–May 2026	NCT04848753	III	500	ESCC	Arm1 : Toripalimab+CRT→Surgery Arm2 : Placebo+CRT→Surgery	DFS
June 2019–October 2023	NCT03957590	III	316	ESCC	Arm1 : Tirelizumab+CRT→Surgery Arm2:Placebo+CRT→Surgery	PFS

EC, esophageal cancer; ESCC, esophageal squamous cell carcinoma; EAC, esophageal adenocarcinoma; EGA, esophagogastric junction carcinoma; GA, gastric adenocarcinoma; DFS, disease-free survival; pCR, pathological complete remission; MPR, major pathological remission; CRT, chemoradiotherapy; CT, chemotherapy; ORR, objective response rate; PFS, progression-free survival.

## Combination of EGFR Inhibitor

EC is a tumor type with a generally high expression of epidermal growth factor receptor (EGFR), and EGFR overexpression is closely related to tumor invasion, metastasis, and poor outcome of EC (85, 86). Theoretically, the prognosis of EC patients can be improved by anti-EGFR strategies. However, the contribution of anti-EGFR chimeric monoclonal antibody such as cetuximab to the treatment of resectable EC remains controversial. Many clinical studies (**Table 2**) attempted to demonstrate the therapeutic effects of EGFR inhibitor combined with radiotherapy and chemotherapy (sequential or concurrent) in the preoperative treatment of locally advanced EC. Based on these small sample studies, it is almost impossible to draw firm conclusions from the available data. The phase III trial of SAKK 75/08 showed that the addition of the EGFR inhibitor cetuximab to CRT improved LRFS particularly with ESCC. However, the addition of EGFR inhibitor seemed to increase toxicity. The use of targeted therapy in resectable EC needs further confirmation.

**Table 2:** Summary of studies using anti-EGFR monoclonal antibodies with chemoradiation for resectable locally advanced esophageal cancer.

Author	Phase	Histology (n)	Treatment Strategy	pCR	OS
Baruch Brenner et al. (87)	IB/II	EAC: 39 ESCC: 25	Cisplatin+5FU + Cetuximab→Surgery	EAC: 20% ESCC: 55%	EAC: 5 yr 25% ESCC: 5 yr 58%
SAKK 75/08 Ruhstaller et al. (88)	III	EAC: 195 ESCC: 105	Arm1 : Cisplatin + Docetaxel→CRT+Cetuximab→Surgery→Cetuximab Arm2 : Cisplatin + Docetaxel→CRT→Surgery	N/A	Arm1: 61.2 mos Arm2: 36 mos
ACOSOG Z4051 Lockhart et al. (89)	II	EAC: 70	Docetaxel+Cisplatin + Panitumumab+RT→Surgery	33%	19.4 mos
HOGG05-92 Carlos et al. (90)	II	EAC: 30 ESCC: 9	Cetuximab+RT→Surgery	EAC: 28% ESCC: 67%	N/A
Vita et al. (91)	II	EAC: 13 ESCC: 28	FOLFOX-4+RT+Cetuximab→Surgery	27%	17.3 mos
Lee et al. (92)	II	EAC: 16 ESCC:3	Irinotecan+Cisplatin + Cetuximab+RT→Surgery	EAC: 16% ESCC: 67%	31 mos

EGFR, epidermal growth factor receptor; EC, esophageal cancer; ESCC, esophageal squamous cell carcinoma; EAC, esophageal adenocarcinoma; pCR, pathological complete remission; CRT, chemoradiotherapy; PFS, progression-free survival; yr, year; mos, months; FOLFOX-4, Folinic acid, Fluorouracil, and Oxaliplatin; N/A, Not Available or Not Relevant.

## Conclusion

With the rapid development of immunotherapy, EC has brought about a shift in management strategy from single therapy to multidisciplinary regimens. Nevertheless, nCRT before surgery is the standard treatment for unresectable locally advanced ECs. However, nCRT itself has room for further in-depth and extensive discussion on the above-mentioned issues. The ability to select subjects who would benefit from nCRT before surgery is of the essence to the clinical decision-making and would accelerate individualized precision therapy.

## Author Contributions

DH and SH were responsible for writing and organizing articles. QZ and JD carried out literature search. HS, WH, and BL were responsible for the article guidance and revision of the manuscript. All authors contributed to the paper and agreed the submitted version.

## Conflict of Interest

The authors declare that the research was conducted in the absence of any commercial or financial relationships that could be construed as a potential conflict of interest.

## Publisher’s Note

All claims expressed in this article are solely those of the authors and do not necessarily represent those of their affiliated organizations, or those of the publisher, the editors and the reviewers. Any product that may be evaluated in this article, or claim that may be made by its manufacturer, is not guaranteed or endorsed by the publisher.
